# Eosinophils in Fungus-Associated Allergic Pulmonary Disease

**DOI:** 10.3389/fphar.2013.00008

**Published:** 2013-02-01

**Authors:** Sumit Ghosh, Scott A. Hoselton, Glenn P. Dorsam, Jane M. Schuh

**Affiliations:** ^1^Department of Veterinary and Microbiological Sciences, North Dakota State UniversityFargo, ND, USA

**Keywords:** allergic asthma, inflammation, eosinophils, fungus

## Abstract

Asthma is frequently caused and/or exacerbated by sensitization to fungal allergens, which are ubiquitous in many indoor and outdoor environments. Severe asthma with fungal sensitization is characterized by airway hyperresponsiveness and bronchial constriction in response to an inhaled allergen that is worsened by environmental exposure to airborne fungi and which leads to a disease course that is often very difficult to treat with standard asthma therapies. As a result of complex interactions among inflammatory cells, structural cells, and the intercellular matrix of the allergic lung, patients with sensitization to fungal allergens may experience a greater degree of airway wall remodeling and progressive, accumulated pulmonary dysfunction as part of the disease sequela. From their development in the bone marrow to their recruitment to the lung via chemokine and cytokine networks, eosinophils form an important component of the inflammatory milieu that is associated with this syndrome. Eosinophils are recognized as complex multi-factorial leukocytes with diverse functions in the context of allergic fungal asthma. In this review, we will consider recent advances in our understanding of the molecular mechanisms that are associated with eosinophil development and migration to the allergic lung in response to fungal inhalation, along with the eosinophil’s function in the immune response to and the immunopathology attributed to fungus-associated allergic pulmonary disease.

## Introduction

Increasing recruitment of eosinophils into affected tissues is a cardinal feature of allergic disease. In allergic asthma, the Th2-mediated immune response orchestrates the production of cytokines and chemokines that coordinate to provide an increase in the number of eosinophils that are produced in the bone marrow to travel through the circulatory system to the lung in response to an inhaled allergen challenge. In recent decades, the prevalence of asthma in the U.S. and other industrialized countries has dramatically increased (Lindell et al., 2008). In 2009, asthma afflicted 8.2% of adults and children in the U.S., 24.6 million persons (Nassenstein et al., 2005; Umetsu and DeKruyff, 2006; Knutsen et al., 2012). In the context of asthma, sensitization to fungi presents a severe clinical scenario that is difficult to treat, accounting for a disproportionately large number of emergency center visits and hospitalizations (Knutsen et al., 2012). Fungal avoidance strategies are often impractical, since fungal spores are ubiquitous in many indoor and outdoor environments and may be found at any time of year. Airway inflammation, marked by a robust eosinophilia, exacerbates asthma symptoms and activates structural cells, which over time changes the architecture of the lung. Metaplasia of the bronchial epithelial layer to mucus-producing goblet cells results in mucus-obstructed airways, and increases in both airway smooth muscle and peribronchial fibrosis often results in significant loss of pulmonary function.

Allergic asthma arises as a result of an immune response triggered by the inhalation of (often) non-infectious environmental antigens. For this reason, respiratory allergies have been classified by many as aberrant immune responses precipitated by a poorly educated immune system or by cross-reactivity to allergens that are similar to host proteins. In their role in the lung, eosinophils, the granulocytes most often associated with allergic asthma, are frequently maligned as participants in the pathogenesis of allergic lung disease. However, recent research suggests that one of the important roles of the eosinophil may be in their ability to carry out important immune functions in the lumen of the airway, a compartment that is not readily accessible by many other cell types. Thus, the eosinophil may be utilized as an antifungal mechanism to prevent infection. This review focuses on the current understanding of the molecular mechanisms that are associated with eosinophil activation, recruitment to the lung, and the function of these multi-factorial granulocytes in allergic/fungus-associated allergic pulmonary disease.

## Development of Eosinophilia in Fungal Asthma

**Figure 1:**
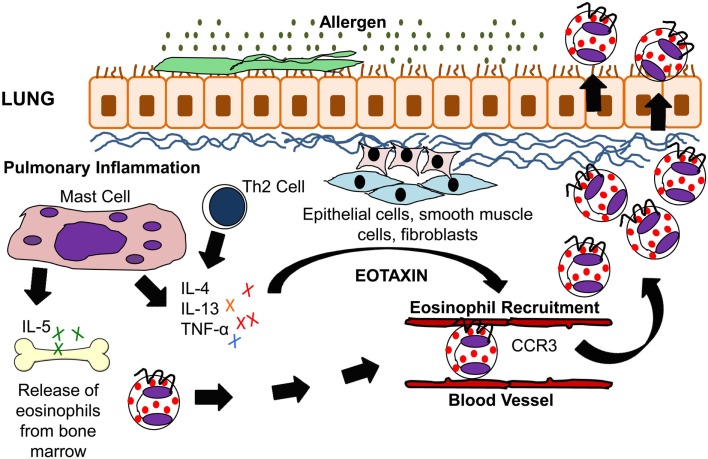
**IL-5 and eotaxin-induced eosinophil recruitment in allergic asthma**. Inhaled allergens activate Th2 lymphocytes and mast cells to produce the cytokines IL-4, IL-5, IL-13, and TNF-α. These cytokines stimulate lung epithelial cells, fibroblasts, and smooth muscle cells to produce eotaxin. IL-5 modulates eosinophil migration from the bone marrow through its action on eotaxin and Th2 cells. Eotaxin on the other hand modulates eosinophil homing to the lung tissue via CCR3 which is present on eosinophils.

Eosinophils develop and mature in the bone marrow from CD34^+^ pluripotent progenitor cells under the influence of interleukin-3 (IL-3) and granulocyte macrophage colony stimulating factor (GM-CSF), with Interleukin-5 (IL-5) acting as a late differentiation factor (Figure 1). Eosinophil differentiation is induced by the synchronized actions of the transcription factors GATA-1 (a zinc finger family member), PU.1 (an Ets family member), and CCAAT/enhancer-binding protein (C/EBP) family members (Hirasawa et al., 2002; McNagny and Graf, 2002; Trivedi and Lloyd, 2007). The contribution of other transcription factors and subtypes may also be important in eosinophil development but is less well established (McNagny and Graf, 2002).

Even though GATA-1, PU.1, and C/EBP are expressed by a variety of hematopoietic lineages, it is their expression and coordination with a unique cytokine and growth factor cocktail that results in the selective development of eosinophils. IL-3, GM-CSF, and IL-5 are associated with the development of various cells of the myeloid lineage and signal through receptors that share a common β-chain but have cytokine-specific α-chains (Sanderson, 1992; Uhm et al., 2012). All of the three cytokines play a central role in eosinophil development, endothelial adhesion, activation, and survival in fungal asthma. They act upon progenitor cells within the bone marrow as well as mature cells in the periphery.

Eosinophils are typically released to the peripheral blood as fully differentiated mature cells. In non-atopic humans, eosinophils make up less than 1% of the peripheral leukocytes in the blood. In allergic conditions, newly released eosinophils circulate in the blood for a short period before homing preferentially to the lung, skin, and gut mucosa using eotaxin chemokine signals and the expressed adhesion marker MAdCAM-1 (Walsh, 1999; Uhm et al., 2012). They can persist in the circulation for 8–12 h and can reside in the tissues for up to 2 weeks through the autocrine production of GM-CSF, which is instigated by the cell’s interaction with the extracellular matrix (ECM; Anwar et al., 1993; Uhm et al., 2012).

In the atopic patient, sensitization of the bronchial mucosa signals an increase in eosinophilopoiesis, which provides a ready pool of mature eosinophils that can be released in response to allergen challenge/fungal challenge (Hogaboam et al., 2000, 2005; Hoselton et al., 2010). Although the number of eosinophils is quite variable, they are elevated in the majority of asthmatics and may reach 30% of the bronchoalveolar lavage (BAL) cells (Wardlaw et al., 2000; Hoselton et al., 2010; Ghosh et al., 2012) that are differentially counted from the airway of asthmatics and 50% of the cells in an induced sputum specimen (Wardlaw et al., 2000). Along with an increase in the number of mature eosinophils in the periphery, there is mounting evidence that CD34^+^ CD45^+^ IL-5Rα eosinophil progenitor cells may be capable of migrating from the bone marrow to the site of allergic inflammation in asthmatic patients (Robinson et al., 1999; Dorman et al., 2005; Menzies-Gow et al., 2007; Murdock et al., 2012). Similar observations have been reported using murine models of fungal allergic asthma showing that 6 h after allergen challenge CD34^+^CD45^+^IL-5Rα^+^ eosinophil progenitor cell populations are elevated in the murine lung (Saito et al., 2002; Johansson et al., 2004; Southam et al., 2005; Murdock et al., 2012; Rosenberg et al., 2013).

## Activation of Eosinophils and Recruitment to the Allergic Lung

Antigen-specific activation of Th2 cells plays an important role in eosinophilic inflammation in allergic asthma, and T cell-deficient mice are protected from lung eosinophilia and airway hyperresponsiveness (AHR; Gavett et al., 1994; Gonzalo et al., 1996). The cytokines IL-4, IL-5, and IL-13, all of which are produced by Th2 cells after fungal allergen challenge, stimulate and enhance the production of eotaxin. Eotaxin then works with the Th2-derived cytokine IL-5 in the selective recruitment of eosinophils (Gutierrez-Ramos et al., 1999; Lloyd et al., 2000; Foster et al., 2001). In a murine model of fungal allergic asthma, eotaxin plays a role in the early recruitment of Th2 cells (Schuh et al., 2002). Fungal studies using an *Aspergillus fumigatus* intratracheal model system have demonstrated IL-17’s central role in driving eosinophilia in Th2-mediated allergic airway inflammation (Schnyder-Candrian et al., 2006; Murdock et al., 2012). IL-17 has also been shown to induce eotaxin-1 expression in human airway smooth muscle cells (Rahman et al., 2006). A summary of eosinophil trafficking in the allergic lung is shown in Figure 1.

Pro-inflammatory cytokines work in coordination with chemokine that are produced at the sites of chronic inflammation to attract mature eosinophils from the bone marrow (Barnes, 2008). The initiation and maintenance of eosinophil migration depends on the cooperative nature of the chemotactic and chemokinetic signals. The receptor profile on the surface of the eosinophil dictates which, if any, chemoattractants will regulate movement and to what extent migration can be induced. By promoting receptor aggregation and the co-localization of downstream signaling mediators, binding of IL-5, IL-3, and GM-CSF primes eosinophil responses to chemoattractants, allowing movement to be initiated by the chemotactic agent (Simson and Foster, 2000; Uhm et al., 2012). In the case of eosinophils, a number of mediators are known to induce eosinophil migration by inducing chemotactic and/or chemokinetic responses in the cell (Table 1).

**Table 1:** **Mediators involved in eosinophil migration**.

Type	Effectiveness	Function	Reference
**CYTOKINES**			
IL-5	Moderate to high	Primes eosinophils, chemotaxis, chemokinesis, selective, and regulate adhesion pathways	Sanderson (1992), Collins et al. (1995), Mould et al. (1997), Simson and Foster (2000), and Barnes (2008)
IL-3 and GM-CSF	Low	Chemotaxis and increase expression of IL-5R Non-selective	Sanderson (1992), Mould et al. (1997), Simson and Foster (2000), Barnes (2008), and Boldajipour et al. (2008)
IL-17	Low		Murdock et al. (2012)
**CHEMOKINES**			
Eotaxins	High	Selective and regulates adhesion pathways	Collins et al. (1995), Mould et al. (1997), Simson and Foster (2000), Borchers et al. (2002), and Paplinska et al. (2007)
MIP-1α	High	Primes eosinophils and non-selective	Schweizer et al. (1996), Simson and Foster (2000), Conti and Digioacchino (2001), Kayaba and Chihara (2001), and Magalhaes et al. (2009)
RANTES	Moderate	Non-selective	Schweizer et al. (1996), Simson and Foster (2000), Conti and Digioacchino (2001), and Borchers et al. (2002)
MCP-3	High	Non-selective	Hansel et al. (1993), Rozell et al. (1996), Schweizer et al. (1996), and Simson and Foster (2000)
IL-8	Low	Non-selective	Hansel et al. (1993) and Hamid and Tulic (2009)
**LIPIDS**			
PAF	Moderate to High	Non-selective	Morita et al. (1989), Hwang (1990), Simson and Foster (2000), Kato et al. (2004), and Rosenberg et al. (2007)
LTB4	Low	Non-selective	Morita et al. (1989), Munoz et al. (1997), Simson and Foster (2000), and Rosenberg et al. (2007)
LTE4	Low	Non-selective	Morita et al. (1989), Munoz et al. (1997), Simson and Foster (2000), Bandeira-Melo and Weller (2003), and Rosenberg et al. (2007)
**FUNGAL COMPONENTS**			
Chitin	Low	Non-selective	Van Dyken et al. (2011)
**ANAPHYLATOXINS**			
C3a	Moderate to high	Selective	Daffern et al. (1995), Discipio et al. (1999), and Discipio and Schraufstatter (2007)
C5a	Moderate to high	Non-selective	Discipio et al. (1999), Guo and Ward (2005), and Discipio and Schraufstatter (2007)

### IL-5

Interleukin-5 is secreted by mast cells and T cells in the late phase of the inflammatory response and plays a key role in the recruitment of eosinophils from the bone marrow, amplifying the chemoattractant potential of chemokines in the tissues. IL-5 primes eosinophils and amplifies intracellular signaling systems coupled to chemokine receptors. The crosstalk between signal transduction molecules used in these processes serves to generate distinct and/or amplified migratory responses (Simson and Foster, 2000). IL-5 is elevated in fungal allergy and plays a critical role in the differentiation, proliferation, and maturation of eosinophils in the bone marrow (Templeton et al., 2010). Recent studies have also shown a role of epithelial cell-derived cytokine thymic stromal lymphoprotein (TSLP), IL-25, and IL-33 in promoting eosinophilia by inducing IL-5 production (Rosenberg et al., 2013).

The IL-5 receptor, IL-5R, is expressed only on eosinophils and basophils (Robinson et al., 1999; Christodoulopoulos et al., 2000; Menzies-Gow et al., 2003, 2007). The expression of IL-5R on eosinophils is closely regulated and depends upon the activation state and anatomical location of the cell. For example, human umbilical cord-derived CD34^+^ cells stimulated with IL-3, IL-5, and GM-CSF leads to the up-regulation of IL-5Rα; an important step in eosinophil lineage commitment (Tavernier et al., 2000). However, its expression is downregulated on mature human eosinophils when treated the same cocktail of cytokines (Gregory et al., 2003). Although the receptors are found on a wider range of cells, administration of IL-3 and GM-CSF has been shown to promote eosinophil production in animal models, as well as in clinical trials (Sanderson, 1992; Ueno et al., 1994; Takamoto and Sugane, 1995).

Furthermore, transgenic mice that constitutively express IL-5 in the lung epithelium develop an accumulation of eosinophils and pathologic changes including goblet cell hyperplasia, epithelial hypertrophy, and AHR even in the absence of antigen challenge (Lee et al., 1997).

### Signal transduction through chemokine receptors

Signal transduction initiated by chemoattractant receptors serves to generate distinct and/or amplified cellular responses (Simson and Foster, 2000). The ability of eosinophils to generate unique cellular responses lies in their activation of their receptor-mediated pathways. The GTPases, which belong to the Ras and Rho families, appear to play an important role at several critical checkpoints in eosinophil development and function (Hall, 1998; Henning and Cantrell, 1998; Muessel et al., 2008), including an integral role in shape, receptor aggregation, and cell migration.

Chemokines signal through G protein-coupled receptors (GPCRs) on the eosinophil’s surface. Three chemokine receptor subgroups are currently recognized – CCR, CXCR, and CX_3_CR1 – which recognize chemokines of the corresponding family. However, in most cases, chemokine receptors recognize more than one chemokine in that family and several chemokines bind to more than one receptor, although this ostensible promiscuity and redundancy may be limited by spatial and temporal regulation of these molecules (Baggiolini, 1998; Simson and Foster, 2000; Ono et al., 2003). In contrast to chemokine receptors, members of the cytokine receptor family consist of cell surface glycoproteins (Bagley et al., 1997; Simson and Foster, 2000; Le et al., 2004). These receptors display similar structural and functional properties. The receptors for IL-5, IL-3, and GM-CSF share structural similarities, although upon ligand binding the IL-5 and IL-3 receptors oligomerize while that of GM-CSF exists as a preformed complex (Simson and Foster, 2000; Ono et al., 2003).

Eosinophils express receptors for the CC chemokine family (Ponath et al., 1996; Borchers et al., 2002). Although many chemokines appear to be highly redundant and their receptors to be promiscuous, only three eosinophil chemotactic cytokines are known to interact with eosinophils. The eotaxins 1–3 signal on eosinophils through the seven-transmembrane receptor CCR3 (Conroy and Williams, 2001; Liu et al., 2006; Rosenberg et al., 2007). Eotaxin acts via the chemokine receptor CCR3 on eosinophils to stimulate the selective recruitment of these cells from the airway micro-vessels into the lung tissue (Pope et al., 2005). Animal models show a fundamental and synergistic role for IL-5 and eotaxin in the migration of eosinophils under basal conditions (Matthews et al., 1998), as well as during fungal allergy (Mould et al., 1997; Matthews et al., 1998; Schuh et al., 2002). Subcutaneous administration of IL-5 induces a concentration-dependent eosinophilia in mice (Palframan et al., 1998).

Eotaxin is expressed basally, but fungal challenge leads to an early increase in production and recruitment of eosinophils in allergic mice (Garcia-Zepeda et al., 1996; Lamkhioued et al., 1997; Matthews et al., 1998; Hoselton et al., 2010; Samarasinghe et al., 2010, 2011a,b). The cytokines produced during the early phase response such as IFN-γ, TNF-α, and IL-1β may regulate the production of eotaxin from endothelial cells which in turn promotes blood and tissue eosinophilia in the late phase response (Cook et al., 1998).

In addition to the eotaxins, eosinophil migration toward an increasing chemokine gradient may be elicited with other CC chemokines including CCL2/MCP-1 (monocyte chemotactic protein-1), CCL3/MIP-1α (macrophage inflammatory protein-1α), CCL5/RANTES (regulated upon activation, normal T cell expressed, and secreted), and CCL7/MCP-3. Although none of these is considered to be a specific eosinophil chemoattractant (Ponath et al., 1996; Schuh et al., 2003), each may play a role in the changing environment of the allergic lung as disease develops. Blockade of eotaxin using neutralizing antibodies (Abs) and single eotaxin-KO (Rothenberg et al., 1997) animals have revealed a significant, yet incomplete, reduction in eosinophilic inflammation (Rothenberg et al., 1997; Uhm et al., 2012). For example, the combined actions of the CC chemokines RANTES, MCP-1, and MCP-5, led to the development of OVA-induced lung eosinophilia (Gonzalo et al., 1998). Although it interacts non-specifically with eosinophils, neutralization of RANTES completely abolished OVA-induced lung eosinophilia (Gonzalo et al., 1998). Eosinophils express the chemokine receptor CCR1, which binds MIP-1α, RANTES, and MCP. There is an increase in the mRNA expression of MCP-1, RANTES, and MIP-1α (Alam et al., 1996; Khalid et al., 2008) after allergen/fungal challenge and MIP-1α blockade has been shown to reduce lung eosinophils by 20% (Holgate et al., 1997; Gonzalo et al., 1998). In addition to CCR1 and CCR3, the CCL1/CCR8 axis has also been shown to preferentially induce the recruitment of eosinophils to the lung following allergen challenge (Lloyd and Rankin, 2003). These data demonstrate that CCR1 and other chemokine receptors may also be an important target in blocking eosinophil responses.

### IL-5/eotaxin synergism for cell recruitment

Eotaxin and IL-5 act co-operatively to regulate eosinophil homing and tissue accumulation (Collins et al., 1995; Choi et al., 2003). Murine studies using IL-5 KO mice have shown that subcutaneous administration of eotaxin alone is insufficient to induce tissue eosinophilia. Tissue eosinophilia could only be restored in these mice by administration of intravenous IL-5 for 72 h (Palframan et al., 1998). Other studies have shown that eotaxin plays an important role in initiating both blood and tissue eosinophilia in the early phase of allergic inflammation (Schuh et al., 2002), while IL-5 is essential for eotaxin-induced tissue eosinophilia (Collins et al., 1995; Rothenberg et al., 1996; Mould et al., 1997).

Following eotaxin activation, a series of events are triggered in the eosinophil, including calcium mobilization, CD11b up-regulation, mitogen-activated protein kinase (MAP-kinase) activation, RhoA/ROCK pathway activation, reactive oxygen production, actin polymerization, and a rapid change in shape which is associated with cell migration/chemotaxis and/or granule release (Schmitz et al., 2000; Conroy and Williams, 2001; Sahai and Marshall, 2003; Paplinska et al., 2007; Muessel et al., 2008).

Incubating human eosinophils with IL-5 increases their migratory response to Platelet-activating factor (PAF), Leukotriene B4 (LTB4), Vasoactive intestinal peptide (VIP), and Formyl-Methionyl-Leucyl-Phenylalanine (FMLP), while having no effect on neutrophils (Numao and Agrawal, 1992; Sehmi et al., 1992; El-Shazly et al., 2000). Therefore, the presence of these mediators in the asthmatic lung may contribute to eosinophil migration in the presence of IL-5 (Trivedi and Lloyd, 2007) and these mediators have been shown to have stimulatory and chemoattractant properties.

### Adhesion molecules

Interleukin-5 and eotaxin cooperate to regulate eosinophil homing and tissue accumulation in allergic/fungal asthma by regulating adhesion pathways used by this leukocyte (Rothenberg et al., 1996; Schuh et al., 2002; Choi et al., 2003). Eosinophils express seven integrin heterodimers (Barthel et al., 2008): α4β1 (CD49d/29), α6β1(CD49f/29), αMβ2 (CD11b/18), αLβ2 (CD11a/18), αXβ2 (CD11c/18), αDβ2(CD11d/18), and α4β7 (CD49d/β7; Georas et al., 1993; Grayson et al., 1998; Tachimoto and Bochner, 2000). Each set of heterodimers interacts with its own ligand which is deposited in ECM or a counter-receptor on another cell. Understanding the function of integrin receptors on a given cell type is complicated by the fact that each integrin may be present in different conformational states, and may have varying levels of expression and clustering on the cell surface (Humphries, 2004; Xiao et al., 2004; Hogg et al., 2011; Long, 2011; Vestweber, 2012). Therefore, the migration of eosinophils to the allergic lung involves a complex interplay of integrin receptors in different states of activation, interacting with a diverse set of ligands on bronchial endothelium and cells within the tissue. The adhesion molecules, particularly the α_4_β_1_ integrin very late antigen (VLA)-4, a ligand for the integrin vascular cell adhesion molecule (VCAM)-1, α_A_β_2_ for ICAM-1, and the P-selectin glycoprotein ligand (PSGL)-1 ligand for P-selectin mediate the migration of eosniophils across epithelial and endothelial barriers (Rosenberg et al., 2007). Eotaxin-1 plays a role in regulating the expression of VLA-4 on eosinophils (Jia et al., 1999; Sung et al., 2000) and studies performed with anti-integrins and blocking Abs for VLA-4 on mice subjected to allergen challenge suggest that this ligand is a crucial component of the eosinophil inflammatory response (Gascoigne et al., 2003; Koo et al., 2003). Because the VLA-4/VCAM-1 interaction promotes the specific adhesion of eosinophils and not neutrophils, several small molecule inhibitors of the VLA-4/VCAM-1 interaction are under exploration as asthma therapeutics (Hagmann, 2004; Okigami et al., 2007). In addition to IL-5 and eotaxin, GM-CSF has also been shown to promote eosinophil migration. Integrins αMβ2 (Mac-1/CD11b) or β2 (CD18) have been shown to play a role in GM-CSF induced eosinophil migration (Muessel et al., 2008). Furthermore, recent studies have shown that in atopic patients there is an increase in the expression of 2B4 (CD244; Munitz et al., 2005; El-Shazly et al., 2011) and CD48 (Munitz et al., 2006) on eosinophils indicating a broader role of these receptors on human eosinophils. Future studies that may involve elucidating the role of these receptors in promoting inflammation in fungal allergy would be of great interest.

Studies using ragweed pollen in both P-selectin- and ICAM-deficient mice have shown a decrease in pulmonary eosinophilia when compared to wild type controls, although eosinophil recruitment was not completely abolished in P-selectin/ICAM-1 double KO mice (Broide et al., 1998). Complete abrogation of eosinophilia was observed in ICAM-1/VCAM-1 double KO mice after allergen challenge (Gonzalo et al., 1996), demonstrating the direct regulation of endothelial-eosinophil interactions governing eosinophilia (Gonzalo et al., 1996).

### Complement proteins, extracellular matrix components, neuropeptides, and other molecules

Platelet-activating factor, VIP, and secretin are capable of inducing chemotaxis of human eosinophils (El-Shazly et al., 1996, 2000; Schweizer et al., 1996; Dunzendorfer et al., 1998). Other eosinophil chemoattractants include lipid mediators such as cysteinyl leukotrienes (LTB_4_ and LTE_4_), bacterial-derived peptide fMLP, and the complement anaphylatoxins C3a and C5a (Rot et al., 1992; Dahinden et al., 1994; Simson and Foster, 2000; Kato et al., 2004).

Complement anaphylatoxins C3a and C5a induce eosinophil migration and extravasation into tissues in allergic/fungal asthma (Humbles et al., 2000; Baelder et al., 2005; Guo and Ward, 2005; Discipio and Schraufstatter, 2007). Although C3a and C5a influence many cell types, they are most well recognized as mediators of leukocyte activation (Hugli, 1986; Jagels et al., 2000). Eosinophils express both C3a and C5a receptors. C3a is highly selective for eosinophil migration, while C5a shows a broader range of cellular actions with an even more potent activation of eosinophil recruitment (Daffern et al., 1995; Discipio et al., 1999). Receptors for C3a and C5a belong to the GPCR family that is characterized by a seven-membrane spanning polypeptide chain, which is functionally associated with a pertussis toxin-sensitive G protein. In granulocytes, this is Gαi (Norgauer et al., 1993; Ames et al., 1996; Roglic et al., 1996; Guo and Ward, 2005). C5a initiates a complex cell signaling system in eosinophils through tyrosine kinase activation of phosphatidylinositol 3 kinase, which induces changes in cellular morphology that are necessary for the cell to migrate along a chemotactic or haptotactic gradient (Norgauer et al., 1993; Ames et al., 1996; Jagels et al., 2000).

Eosinophils survive in the tissues due to an autoregulatory production of GM-CSF as a result of the adhesion of α4 to fibronectin in the extracellular tissue matrix (Mishra et al., 1999). Recent studies by Ohkawara et al. (2000) have shown a role of the glycosaminoglycan hyaluronic acid, which is also a component of the ECM in the activation and survival of eosinophils. Further studies on the role of ECM components in eosinophil migration, chemotaxis, and function in fungal asthma would have important implications for understanding their role in eosinophil activation in health and disease.

In the past decade or so, intensive work in the fields of neuropeptides and immune cells has resulted in accumulating evidence that supports the existence of a neuroimmune axis (Numao and Agrawal, 1992; El-Shazly et al., 2000). Neuropeptides, such as VIP and secretin, are capable of inducing chemotaxis of human eosinophils (Schweizer et al., 1996; Dunzendorfer et al., 1998; El-Shazly et al., 2000). Furthermore, VIP has been shown to induce eosinophil derived neurotoxin (EDN) release in a potency comparable to that induced by platelet-activating factor (El-Shazly et al., 2000). Studies using an *A. fumigatus* inhalational allergic model system have shown that the VIP signaling through its VPAC2 receptor dysregulates or causes significant temporal delays of immune cell recruitment and Th2 polarization (Hoselton et al., 2010; Samarasinghe et al., 2010). *In vivo* experiments using VPAC2-deficient mice in an allergic fungal model have supported the proposition that the Th2 phenotype is induced by VPAC2 signaling, as mice deficient for VPAC2 showed a 75% reduction in the recruitment of eosinophils to the airway lumen (Samarasinghe et al., 2010, 2011a). Further studies to elucidate the mechanism of eosinophil migration using an autocrine VIP/VPAC2 signaling loop and its effect on chemotaxis would be of great interest.

A similar observation with VIP and eosinophil migration has been reported recently in an allergic rhinitis model (El-Shazly et al., 2013). Eosinophils infiltrated in the allergic nasal tissue have been shown to express high levels of VIP. Furthermore, eosinophil treatment with VIP has been reported to up-regulate the expression of CRTH2 (CD294) on human eosinophils and total CRTH2 protein (El-Shazly et al., 2013). This phenomena was shown to be independent of VPAC1 and VPAC2 suggesting a possible role of CRTH2 in eosinophil migration. However, the role of this receptor in eosinophil migration in the context of fungal allergy remains to be elucidated.

## Fungus-Associated Pulmonary Allergy and Pathology

### Development of allergic fungal respiratory disease

Allergic fungal asthma is a chronic disease that is important from both a personal and public perspective. AHR, inflammatory infiltrates, smooth muscle increases, and fibrotic remodeling of the bronchial architecture are features of allergic fungal asthma. Sensitization and colonization by fungal species often results in chronic architectural changes in the lung, causing long-term morbidity (Denning et al., 2006; Knutsen and Slavin, 2011), reduced productivity and quality of life, as well as increased costs associated with medical treatment. Epidemiological studies in the U.S. and Europe have associated mold sensitivity to *Alternaria alternate* and *Cladosporium herbarum* with the development, persistence, and severity of asthma (Knutsen et al., 2012). In addition, sensitivity to *A. fumigatus* has been associated with severe persistent asthma in adults (Knutsen et al., 2012). Severe asthma with fungal sensitization (SAFS) is a new designation in pulmonary diagnostics and treatment (Denning et al., 2006) and experimental models using *A. fumigatus* are being used to explore the course and mechanisms at play in fungal interactions.

The majority of fungal spores counted from outdoor air samples are from the phyla Ascomycota or Basidiomycota (Horner et al., 1995). The most commonly studied fungal allergens are *Aspergillus*, *Alternaria*, *Botrytis*, *Cladosporium*, *Epicoccum*, *Fusarium*, and *Penicillium* species (Knutsen et al., 2012). In a recent study, over 40% of children who had failed combination therapy with high dose inhaled corticosteroids and long-acting beta agonists were diagnosed with SAFS (Vicencio et al., 2012). In this study, many children (65%) displayed antibody specificity to more than one fungal species, with *Aspergillus* (81.2%) and *Alternaria* (68.8%) species being the most commonly associated with sensitivity (Vicencio et al., 2012). Conidia (spores) are present in the outdoor environment throughout the year in many environments and frequently exceed the pollen population by 100- to 1000-fold (Knutsen et al., 2012). Spores and fungal fragments found in indoor environments originate from fungi present outdoors and from fungi that grow in moist indoor environments such as damp basements (Aukrust, 1979).

*Aspergillus fumigatus* is distributed widely in the environment. It is a saprophytic mold with an important environmental function in carbon and nitrogen cycling (Dagenais and Keller, 2009). As an opportunistic pathogen of plants and animals as well as a prominent sensitizing agent in allergic respiratory diseases, *A. fumigatus* is among the most well recognized and best studied fungal species of the total estimated 3.5–5.1 million that are predicted from high throughput environmental screening (O’Brien et al., 2005). Its hydrophobic spores are readily dispersed in the environment and, when inhaled, are small enough to navigate the airways of the lung far beyond the barriers of the ciliated epithelium (Latge, 1999). The growth habit and physical characteristics of *A. fumigatus* make it an opportunistic pathogen of humans and an ideal carrier of aeroallergens. Cellular innate (neutrophil- and macrophage-mediated) and adaptive (Th1-mediated) immune responses protect against infection by *Aspergillus* in a normal lung (Grazziutti et al., 1997; Traynor and Huffnagle, 2001; Beck et al., 2006; Murdock et al., 2011), but *A. fumigatus* can also induce or exacerbate allergies of the upper and lower airways, and exposure to *Aspergillus* can result in invasive aspergillosis (IA) in immunocompromised patients.

Sensitization to fungal species arises from a combination of genetic and environmental factors, along with certain characteristics of the allergen itself. Both indoor and outdoor environmental exposure has been associated with asthma exacerbations (Pongracic et al., 2010). Some studies have demonstrated a correlation between visible mold growth in homes and asthma episodes in children (Bundy et al., 2009; Karvonen et al., 2009). Both allergic rhinitis and asthma have been associated with exposure to fungal contamination in homes (Park et al., 2008). A recent study involving a quantitative meta-analysis of 33 epidemiological studies has shown an increase of 30–50% in adverse respiratory health outcomes in occupants because of dampness and mold exposure (Fisk et al., 2007). Furthermore, recent reviews from the United States, Europe, and the World Health Organization affirm that a damp indoor environment is a factor in asthma development (Mendell et al., 2011).

*Aspergillus*-induced asthma is characterized by increases in mature eosinophils and their progenitors within the bone marrow, blood, and bronchi. In asthmatic patients, eosinophils generate a variety of pro-inflammatory mediators that can disrupt epithelial integrity and inflict damage to the ECM. In addition, they stimulate the degranulation of mast cells and basophils (Reed, 1994; Makino and Fukuda, 1995; Pearlman, 1999; Hogaboam et al., 2003; Clark et al., 2004; Williams, 2004; Kariyawasam and Robinson, 2007; Hoselton et al., 2010; Venge, 2010; Samarasinghe et al., 2011a,b). For these reasons, eosinophils represent potential effector cells in the pathogenesis of allergic fungal asthma.

### Pathology of fungal allergic asthma

Sensitization to *Aspergillus* is common in atopic individuals and *A. fumigatus* is responsible for approximately 16–38% of *Aspergillus*-related illness in humans (Schwartz et al., 1978; Maurya et al., 2005). In asthmatic individuals, *Aspergillus* sensitization, or allergic bronchopulmonary aspergillosis (ABPA), is characterized by exacerbations of asthma, recurrent transient chest radiographic infiltrates, expectoration of thick mucus plugs, blood and pulmonary eosinophilia, and increased total serum and fungus-specific IgE levels. ABPA is the most common form of allergic bronchopulmonary mycosis (ABPM) although other fungi, including *Candida*, *Penicillium*, and *Curvularia* species, are also implicated. Balls of fungus called aspergillomas may form following repeated exposure to conidia and target preexisting lung cavities such as the healed lesions in tuberculosis patients. IA is the most devastating of the *Aspergillus*-related diseases, targeting severely immunocompromised patients (Dagenais and Keller, 2009). In immunocompromised patients or those with previous lung damage, *A. fumigatus* can germinate and its growth may invade local blood vessels causing disseminated fungal disease with mortality rates ranging from 40 to 90% (Lin et al., 2001; Dagenais and Keller, 2009).

Some of the symptoms of fungal asthma are familiar: sneezing, coughing, mucus production, AHR. Although the chronic changes in the structure of the airway wall are less obvious, they represent an accumulated dysfunction that can significantly impact a patient’s quality of life (Lacoste et al., 1993; Jeffery, 2001; Agrawal and Shao, 2010; Bellido-Casado et al., 2010; Hoselton et al., 2010). As inflammation plays an important role in most of the symptoms that are associated with fungal asthma, much attention has been focused on delineating the mechanisms of development and persistence of inflammation. Repeated exposure to allergens like *A. fumigatus* results in the accumulation of neutrophils, basophils, and mast cells which are characteristic features associated with the early phase inflammatory reaction (Galli, 2000; Hogaboam et al., 2003; Bosiger and Fehr, 2006; Verstraelen et al., 2008; Hoselton et al., 2010; Amin, 2012), while the late phase reaction is characterized by the accumulation of Th2 lymphocytes (Horwitz and Busse, 1995), B lymphocytes (Horwitz and Busse, 1995; Ghosh et al., 2012), neutrophils (Horwitz and Busse, 1995; Hogaboam et al., 2003), macrophages (Horwitz and Busse, 1995; Hogaboam et al., 2003; Samarasinghe et al., 2011b), basophils (Horwitz and Busse, 1995; Smit and Lukacs, 2006), and eosinophils (Smit and Lukacs, 2006; Campos and Pereira, 2009; Samarasinghe et al., 2011b) in the airway tissue, including the sub-mucosa, epithelium, and airway lumen (Horwitz and Busse, 1995; Hogaboam et al., 2003; Smit and Lukacs, 2006; Verstraelen et al., 2008; Campos and Pereira, 2009; Fahy, 2009; Hamid and Tulic, 2009; Hoselton et al., 2010; Samarasinghe et al., 2011a,b; Ghosh et al., 2012).

### Eosinophil-associated damage in allergic asthma

Eosinophils have a central role in the inflammatory milieu that is established within the asthmatic lung, and primed eosinophils can be further activated by numerous stimuli including GM-CSF, IL-5, and Abs (Capron et al., 1989; Kotsimbos and Hamid, 1997; Adachi and Alam, 1998; Flood-Page et al., 2003; Bartemes et al., 2005). GM-CSF activates and enhances eosinophil functions, such as superoxide production, leukotriene production, phagocytosis of serum opsonized zymosan, and Ig-induced degranulation (Kita, 2011). IL-5, apart from increasing the chemotactic response of eosinophils, also plays a role in superoxide generation, phagocytosis, and immunoglobulin-induced degranulation (Kita et al., 1992).

Eosinophils can also recognize the products of adaptive immunity. Sepharose beads coated with IgG, IgA, and secretory IgA (sIgA) have been shown to stimulate eosinophil degranulation, and sIgA was the most effective among these Igs. The exact mechanism to explain why sIgA is more potent is unknown but eosinophils possess binding sites for the secretory component. Furthermore, interaction with sIgA increases eosinophil pro-inflammatory function (Abu-Ghazaleh et al., 1989; Monteiro et al., 1993; Motegi and Kita, 1998). The role of IgE, which is a hallmark of allergic disease, in mediating eosinophil activation is controversial. Some studies have shown that eosinophils isolated from patients with eosinophilia degranulate in response to anti-IgE antibody (Moqbel et al., 1990) and that a high-affinity IgE receptor is present on eosinophils from patients with eosinophilia and various effector functions are mediated through this receptor (Moqbel et al., 1990; Gounni et al., 1994). Other studies have shown that the number of high-affinity receptors expressed on the surfaces of eosinophils from patients with allergic diseases was minimal and that ligation of FcεRI does not result in eosinophil degranulation (Kita et al., 1999; Seminario et al., 1999). However, the role of high-affinity receptors in mouse models of fungal asthma are still unclear.

Eosinophils are able to release stored mediators via three highly regulated degranulation mechanisms, classical exocytosis, compound exocytosis, and piecemeal degranulation. Degranulation can also occur by cytolysis where the entire contents of the cell are released during rupture (Logan et al., 2003). Classical exocytosis involves the extrusion of single secretory granules but has not been demonstrated in airway tissue (Erjefalt and Persson, 2000). Compound exocytosis involves the fusion of multiple intracellular granules followed by a focused secretion onto the target cell at the site of adhesion (Hafez et al., 2003); while piecemeal degranulation allows the partial and selective release of granule contents by transferring them into small vesicles that are subsequently released by exocytosis. This is demonstrated by the selective release of RANTES from IFN-γ stimulated human eosinophils independently of both major basic protein (MBP) and eosinophil peroxidase (EPO) release (Lacy et al., 1999).

The cytotoxic compounds contained in the granules of eosinophils are capable of killing filarial stages. The eosinophil’s anti-helminthic functions have been recognized since the mid-1970s (Butterworth et al., 1975). The same granules were recognized to be cytotoxic to bronchial epithelium, as well, and have for some time been firmly associated with the immunopathology of allergic asthma (Butterworth et al., 1975). In addition, release of eosinophilic granules increases vascular permeability *in vivo* at physiologic concentrations that are observed in pathological conditions associated with allergic/fungal asthma (Piliponsky et al., 2002; Bloemen et al., 2007). They also activate mast cell release of pro-inflammatory mediators including histamine, eicosanoids, and cytokines (Minnicozzi et al., 1994; Bloemen et al., 2007). Eosinophils produce stem cell factor (SCF) and nerve growth factor (NGF), which further support the growth and survival of mast cells (Piliponsky et al., 2002). Eosinophils also produce the Th2-type cytokines IL-4 and IL-13, which potently stimulate the release of eotaxin and the production of RANTES and MCP-1, further enhancing eosinophil recruitment. Eotaxin can induce respiratory burst and actin polymerization in eosinophils, directly contributing to tissue damage as well as orchestrating the continual recruitment of both eosinophils and T cells (Li et al., 1999). A summary of different mediators released by eosinophils in the allergic lung is delineated in Table 2.

**Table 2:** **Mediators released by eosinophils**.

	Mediator	General function	Reference
Basic granule proteins	Major basic protein (MBP)	Respiratory epithelial desquamation	Frigas et al. (1981) and Gleich (2000)
		M2 receptor dysfunction	Fryer and Jacoby (1998) and Takafuji et al. (1998)
		Mammalian cell and parasite toxicity	O’Donnell et al. (1983) and Piliponsky et al. (2002)
		Stimulation of neutrophils,	Gleich (2000)
		Mast cells, and basophils	Jacoby et al. (1993)
	Eosinophil cationic protein (ECP)	Bronchial Hyperresponsiveness	Gleich (2000) and Weller (2008)
		Leads to bronchoconstriction	Rosenberg et al. (1989) and Weller (2008)
		Respiratory epithelial desquamation	Gleich (2000)
		Cell and parasite toxicity	Zheutlin et al. (1984)
		Generation of radical species	Kay (1988) and Gleich (2000)
		Stimulation of mast cells	Wu et al. (1999) and Matsunaga et al. (2000)
	Eosinophil peroxidase (EPO)	Suppression of lymphocyte response	Takafuji et al. (1998) and Gleich (2000)
		Mast cell and basophil degranulation	Ayars et al. (1989) and Fryer and Jacoby (1998)
		M2 receptor dysfunction	Wu et al. (1999) and Gleich (2000)
		Cell and parasite toxicity	Wardlaw et al. (2000)
		Generation of oxygen radicals	Wu et al. (1999)
Chemokines	CCL2, CCL3, CCL11, CCL5 and IL-8	Migration of monocytes, macrophages, neutrophils, T cells, and eosinophils	Yousefi et al. (1995), Ying et al. (1996), and Nakajima et al. (1998)
		Increased eosinophil survival	Gleich (2000)
		Increased adhesion molecules expression	Weller (2008)
		Airway wall remodeling	Elsner and Kapp (1999)
Cytokines	IL-3, IL-5, IL-9, GM-CSF, IFN-γ, TNF-α, and IL-2	Sustained inflammation	Elsner and Kapp (1999)
	IL-6, IL-4, IL-13, IL-16, IL-17, IL-2, and IL-8	Eosinophil migration, development, and survival	Sanderson (1992) and Weller (2008)
		Increased adhesion molecule expression	Arm and Lee (1992) and Sanmugalingham et al. (2000)
		Airway wall remodeling	Nonaka et al. (1995) and Woerly et al. (2002)
			Rand et al. (1991) and Minshall et al. (2000)
Lipids	Cysteinyl leukotrienes, PAF, PGD_2_, and PGE_2_	Increased mucus secretion	Kupczyk and Kuna (1999) and Weller (2008)
		Increased vascular permeability	Kupczyk and Kuna (1999) and Gleich (2000)
		Activation of eosinophils, mast cells, basophils, neutrophils, and platelets	Kupczyk and Kuna (1999) and Gleich (2000)
		Smooth muscle cell contraction	Kupczyk and Kuna (1999) and Gleich (2000)
		Increased adhesion molecules expression	Kupczyk and Kuna (1999) and Gleich (2000)
		Chemotaxis of eosinophil and neutrophil	Kupczyk and Kuna (1999) and Weller (2008)

Airway hyperresponsiveness refers to the increased ability of the airways to narrow after exposure to non-specific stimuli. It is a classical feature of *Aspergillus*-induced allergic asthma, and AHR severity correlates with the severity of the disease (Hogaboam et al., 2003; Boutet et al., 2007; Dagenais and Keller, 2009; Ramaprakash et al., 2009; Samarasinghe et al., 2010, 2011a,b). Similarly, eosinophilic inflammation is the characteristic feature associated with allergic asthma and it broadly correlates with disease severity (Hogaboam et al., 2003; Boutet et al., 2007; Dagenais and Keller, 2009; Ramaprakash et al., 2009; Samarasinghe et al., 2010, 2011a,b). Eosinophils that are recruited and activated after allergen challenge are believed to contribute to AHR by the direct release of pro-inflammatory mediators and by interaction with other cell types. In addition, eosinophils indirectly contribute to the development of AHR by the induction of mast cell and basophil degranulation, leading to the local release of prostaglandins, leukotrienes, and histamines, all of which can induce AHR (Kay, 1983). Many studies support this long-standing view of the eosinophils as a central effector in allergic airway disease. In C57BL/6J murine model of allergic asthma, thorough depletion of eosinophils using an antibody against CCR3 results in a down regulation of AHR with observed changes in the number of other cell types (Justice et al., 2003).

The impact of eosinophils on the activation of Th2 cells is another way that they contribute to the ongoing allergic lung response. While the Th2-type cytokine IL-13 can induce AHR independently of eosinophilia (Grunig et al., 1998), ablation of eosinophils in a IL-5/eotaxin double knockout system abolishes AHR by reducing the ability of T cells to produce IL-13 (Mattes et al., 2002).

Airway remodeling refers to structural changes in the asthmatic airways which occur as a result of dysfunctional repair processes within the lung. It is characterized by the increased deposition of ECM proteins such as collagen I and tenascin within the reticular basement membrane and bronchial mucosa, increases in airway smooth muscle mass, and goblet cell hypertrophy and hyperplasia (Samarasinghe et al., 2010, 2011a,b; Girodet et al., 2011). Airway remodeling may contribute to AHR and fixed airway flow obstruction and also contribute to the loss of lung function over time (Kariyawasam and Robinson, 2005).

Eosinophils release a number of mediators that have been associated with airway remodeling. The essential role of eosinophils in airway remodeling was first described by a study in which eosinophils were genetically ablated in mice by the deletion of the high-affinity GATA-binding site in the GATA-1 promoter (McMillan and Lloyd, 2004). After a period of prolonged allergen challenge, wild type mice exhibited the prominent features of airway remodeling, namely increased sub-epithelial deposition of collagen together with airway smooth muscle cell hypertrophy and proliferation, but these features were abrogated in double knockout GATA mice (Humbles et al., 2004).

**Figure 2:**
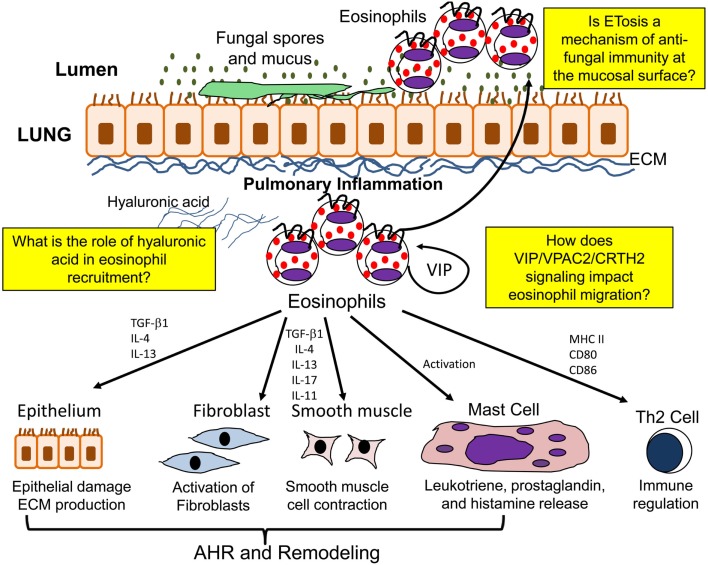
**Function of eosinophils in the allergic lung**. In the allergic lung eosinophils are activated to release a number of mediators which may contribute to airway hyperresponsiveness (AHR), airway remodeling, immunomodulation, and ETosis.

Eosinophils additionally contribute to airway remodeling and fibrosis in allergic/fungal asthma by synthesizing a number of pro-fibrotic mediators. Eosinophils are thought to be an important source of the pro-fibrotic cytokine TGF-β (Minshall et al., 1997; Ohkawara et al., 2000; Cho et al., 2004; Rosenberg et al., 2013). Studies have shown that eosinophils release TGF-β in response to low molecular mass HA and that IL-4 and IL-5 can stimulate eosinophils to release TGF-β *in vitro* (Elovic et al., 1998; Ohkawara et al., 2000). However, other cell types also have the capability to produce TGF-β (Boxall et al., 2006). TGF-β is able to induce ECM protein production, and also contributes to the accumulation of fibroblasts below the reticular basement membrane by stimulating fibroblast proliferation (Fine and Goldstein, 1993; Richter et al., 2001; Kenyon et al., 2003; Doherty and Broide, 2007). Eosinophils also promote the differentiation of myofibroblasts from resident fibroblasts (Masur et al., 1996) and also from circulating precursor cells known as fibrocytes (Mori et al., 2005). In addition, the differentiation of myofibroblasts into smooth muscle cells and their proliferation may also be governed by TGF-β (Wicks et al., 2006). IL-5 KO mice have significantly reduced BAL eosinophils and airway remodeling in a model of chronic allergen challenge (Cho et al., 2004; Tanaka et al., 2004). Both studies show a role of eosinophil derived TGF-β in the propagation of airway remodeling. Furthermore, administration of an anti-TGF-β antibody in sensitized mice followed by allergen challenge prevented the progression of airway remodeling without altering inflammation (McMillan et al., 2005). Treatment of asthmatic patients with an anti-IL-5 antibody reduces the deposition of ECM proteins within the lung with a reduction in BAL TGF-β (Flood-Page et al., 2003). However, the precise role of TGF-β derived from eosinophils is complicated by the fact that the TGF-β may induce the expression of other fibrotic factors such as plasminogen activator inhibitor while being able to act in an either synergistic or antagonistic manner with other factors such as epidermal growth factor (Hara et al., 2001). A summary of eosinophil function in the allergic lung is shown in Figure 2.

### Effect of anti-eosinophil treatments

The fact that a number of chemotherapeutic agents that are effective in alleviating asthma symptoms reduce tissue eosinophilia in allergic asthma has cemented the role of eosinophils as immunopathologic in the minds of many scientists and clinicians. Glucocorticoids induce a marked eosinopenia when given orally, and both oral and inhaled glucocorticoids reduce tissue eosinophilia (Wardlaw et al., 2000). Oral prednisolone has been shown to cause an amelioration of sputum eosinophilia and ECP level in patients with severe disease exacerbations, which correlates with improvement in lung function (Claman et al., 1994). Leukotriene antagonists modestly reduce eosinophilia in the allergic lung (Rothenberg and Hogan, 2006). In addition, cyclosporin and a thromboxane A_2_ antagonist reduce the eosinophil count in asthmatic airways although is not clear if their effect on eosinophils is a major role as they have a wide range of other actions in asthma (Claman et al., 1994; Hoshino et al., 1999; Khan et al., 2000). Omalizumab, a humanized monoclonal antibody which binds to free IgE, has been shown to block the release of inflammatory mediators from mast cells and reduces the infiltration of inflammatory cells, notably eosinophils (Sarinho and Cruz, 2006).

Given the central role of IL-5 in eosinophil development and action, a number of studies have focused on blocking IL-5 and its effect on eosinophilia and the symptoms associated with allergic asthma. Clinical trials have targeted IL-5 using 2 humanized monoclonal Abs, SCH55700 (Schering-Plough Research Institute, Kenilworth, NJ, USA) and mepolizumab (GlaxoSmithKline, Middlesex, UK; O’Byrne et al., 2001; Kay and Klion, 2004). In a randomized, double-blind study of mepolizumab, clinical symptoms of patients with asthma were unaffected despite a dramatic decline in peripheral blood eosinophilia. Most interestingly, despite repeated administration of anti-IL-5 therapy, eosinophils persisted in the lung tissue and in the airway (Kay and Klion, 2004). An independent trial performed with SCH55700 resulted in a similar depletion of peripheral blood eosinophils without improvement in clinical symptoms. These results may be related to complexities of specific disease states (O’Byrne et al., 2001).

Eosinophils have long been reported to produce the cytokine TGF-β in allergic asthma (Leavy, 2008). TGF-β is one of the main effectors involved in tissue remodeling in the asthmatic lung and is overexpressed in the allergic lung (Kay et al., 2004; Leavy, 2008). Potential therapeutic applications that modulate the TGF-β response in fungal asthma may help to elucidate the role of both TGF-β and eosinophils in allergic asthma.

## Mechanisms of Eosinophil-Mediated Immunity

In the context of an allergic respiratory response to an inert agent like pollen, animal dander, or house dust mite, eosinophils have an entrenched identity as instigators and perpetuators of an immunopathologic inflammatory response (Tenscher et al., 1996). Interestingly, recent information examining the conservation of allergen sequence homology across helminth, protozoan, and fungal organismal databases show that minor allergens generally have common homologs among these groups and also may have homologs in common with bacterial or human proteins. However, major allergens – defined as a specific substance that elicits an IgE response in at least 50% of the individuals who are allergic to the complex mixture in which the substance is found – are often unique to the individual helminth, protozoan, or fungal group (Santiago et al., 2012). If, then, allergy is not merely a vestige of cross-reactivity among classes of pathogens or host proteins, what is its role in the adaptive immune response to helminths, protozoans, and/or fungi?

Let us look at the role of the eosinophil, not from an immunopathologic perspective, but from that of an immune effector cell. Eosinophilia is a characteristic of many allergic diseases, and the accumulation and degranulation of these cells in a tissue may contribute to epithelial sloughing. The epithelium turns over quickly in an atopic lung. Murine models of fungal allergic asthma show a marked and dynamic metaplasic phenotype in which nearly 100% of the lining of the large airways is not ciliated columnar cells, but mucus-producing, non-ciliated goblet cells. In an overwhelming inhalation of fungal spores when the innate mechanisms that typically phagocytose and eliminate fungal spores from the airways become inundated, a mechanism by which the columnar epithelium is replaced by sticky, mucus-producing cells may make the most sense.

Recent work shows an important antifungal role for these granulocytes in the context of the pulmonary lumen. Eosinophils have been reported to exert a strong inflammatory response against germinating *A. alternata* resulting in killing of the fungus (Yoon et al., 2008). This phenomena was shown to be mediated by a β-2 integrin adhesion molecule, CD11b which is present on eosinophils and can interact with β-glucan present on the surface of *A. alternata* (Yoon et al., 2008) further suggesting an important antifungal role of these granulocytes.

Eosinophils, frequently associated with chronic allergic conditions and asthma, have molecular receptors that have been implicated in the recognition of *A. fumigatus* components. Targeting eosinophils has proven effective at ameliorating symptoms in patients with severe asthma (Haldar et al., 2009). β-d-glucan, a major component of the fungal cell wall, has been associated with an increased peak expiratory flow variability in children with asthma (Douwes et al., 2000). Dectin-1, which is a receptor for β-d-glucan is present on macrophages, neutrophils, and dendritic cells and it transduces signals to various cell responses with phagocytosis, oxidative burst, and production of inflammatory mediators, including IL-8, IL-6, IL-12, IL-18, and TNF-α (Hohl, 2008; Goodridge et al., 2009). Recent studies report the presence of dectin-1 on human eosinophils, indicating that fungal components can directly activate eosinophils (Goodridge et al., 2009; Kvarnhammar and Cardell, 2012).

Chitin is another component of the fungal cell wall and has been identified as a recognition element capable of initiating immune responses associated with allergy and asthma (Chatterjee et al., 2008; Van Dyken et al., 2011). Increased chitinase levels have been associated with asthma and increased IgE levels (Chatterjee et al., 2008). Furthermore, eosinophils are recruited in response to chitin by a mechanism dependent on the high-affinity LTB4 receptor (Reese et al., 2007). In summary, all the above mentioned studies suggest an important immune effector function of eosinophils in the context of allergic disease.

### Immunomodulation

The recognition of eosinophils as complex immunomodulatory cells has been increasing in recent years. One novel immunomodulatory function of eosinophils is that they can act as antigen presenting cells (APCs) as they express MHC Class II (Koeffler et al., 1980; Lucey et al., 1989). *In vitro*, the expression of MHC Class II is dependent on stimulation of eosinophils by GM-CSF (Lucey et al., 1989). More immediately relevant to *in vivo* disease are a series of observations that eosinophils recovered from sites of allergic or parasitic inflammation expressed MHC Class II (Hansel et al., 1991; Sedgwick et al., 1992; Mawhorter et al., 1993). In patients with asthma, eosinophils isolated from sputum expressed the MHC Class II protein HLA-DR. In mouse models of parasitic infection, MHC Class II was upregulated in eosinophils recovered from sites on infection (Mawhorter et al., 1993). MHC Class II alone, however, is insufficient for professional antigen presentation; the presence of co-stimulatory molecules is necessary for cells to act as professional APCs, as defined by the ability to present antigen to naive T cells, resulting in their activation (Schwartz, 2001). Ohkawara et al. (1996) demonstrated that eosinophils isolated from the peripheral blood of mildly atopic subjects express the co-stimulatory protein CD40. Two major co-stimulatory molecules, CD80 and CD86, have been shown in murine experimental models of allergic lower airway inflammation to be expressed on eosinophils recovered from these sites (Shi et al., 2000; MacKenzie et al., 2001). In addition, antigen loaded murine eosinophils elicited proliferation of T cells *in vitro* that was inhibited by the presence of anti-CD80 and anti-CD86 Abs (Tamura et al., 1996; Shi et al., 2000).

### ETosis

Under conditions of chronic inflammation in allergic asthma, neutrophils along with eosinophils are the first cells to be recruited to inflammatory sites (Baggiolini, 1998; Agrawal and Shao, 2010). Neutrophils, which are the most abundant leukocytes in the blood, use two basic strategies to eliminate microorganisms (Guimaraes-Costa et al., 2012). They can kill microorganisms via phagocytosis, which involves ingestion and killing of microorganisms inside special compartments of the cell. Alternatively, they can kill microorganisms via degranulation, which consists of extravasation of the granular contents to the extracellular milieu. In addition to these two mechanisms, recent studies have identified a new antimicrobial mechanism that neutrophils can use to eliminate microbes. This mechanism was first termed NETosis in reference to the neutrophil extracellular traps that are deployed into the extracellular milieu when DNA-associated proteins are expelled from the cell to eliminate microbes (Brinkmann et al., 2004; Brinkmann and Zychlinsky, 2007; Guimaraes-Costa et al., 2012). NETosis has been observed in many experimental models of fungal and bacterial infections (Urban et al., 2006; Bruns et al., 2010; Guimaraes-Costa et al., 2012). However, it is now recognized that other cell types such as eosinophils and mast cells may also use extracellular traps (von Kockritz-Blickwede et al., 2008; Yousefi et al., 2008). Monocytes and macrophages have also been shown to release ETs but to a lesser extent than that of granulocytes (Webster et al., 2010). Since this general mechanism is now known to be shared by different cell types, the release of ETs was termed as ETosis, meaning death with release of DNA extracellular traps (Guimaraes-Costa et al., 2012).

ETosis seems to be a well-conserved mechanism in eosinophils, as studies have shown extracellular DNA with ECP and MBP in the innate defense mechanism against helminths and bacteria in gastrointestinal infections (Yousefi et al., 2008). The extracellular DNA activity (i.e., ETosis) of intact eosinophils might be particularly crucial against fungi and other pathogens at the surface of the mucosa and may well play an important role in fungal allergic asthma where eosinophils are present in large numbers in the airway lumen and the lung (Clark et al., 2004; Hoselton et al., 2010). Further studies on the role of ETosis in impacting eosinophil function at the mucosal interface would have important implications in understanding the role of ETosis in eosinophil activation in health and disease.

## Conclusion

Observations from experimental animals and asthmatic patients suggest a direct participation of eosinophils in mediating the pathophysiology associated with allergic/fungal asthma, although the mechanisms by which eosinophils contribute to the pathogenesis are rather complicated. Eosinophils have been considered end-stage cells in immunopathology of fungal allergic asthma. However, emerging evidence suggests that eosinophils have a broad range of functions beyond that of a basic granulocyte in allergic asthma and that they promote more than one aspect of respiratory dysfunction which are associated with allergic/fungal asthma. The initiation and maintenance of allergic/fungal asthma relies on the balance between the early and late phase inflammatory response. Hence, the possibility of drugs directed at inhibiting eosinophil migration, activation, or even outright eosinophil ablation might prove to be effective therapeutic strategies. Furthermore, a directed exploration of the factors permitting prolonged survival of eosinophils in tissue, even in the presence of effective IL-5 blockade, might uncover additional eosinophil depletion strategies. Thus, further studies using *in vivo* eosinophil deficient mice and further characterization of the immunobiology of eosinophils *in vitro* will be necessary to answer critical questions concerning the involvement of these leukocytes in fungal asthma. Among the avenues that that might be considered is the possibility by which eosinophils integrate and prioritize the extra-and intracellular signals from the collective actions of cytokines, chemokines, and the role of VIP/VPAC2/CRTH2 signaling and ECM components like hyaluronic acid in eosinophil chemotaxis/migration will allow the development of specific therapeutic targets which can further attenuate specific components of the fungal allergic response. Furthermore, studies involving the recognition of epigenetic factors in regulating the inflammatory genes in fungus-associated pulmonary allergic disease may lead to new therapeutic approaches in the future.

## Conflict of Interest Statement

The authors declare that the research was conducted in the absence of any commercial or financial relationships that could be construed as a potential conflict of interest.
